# Perioperative Predictors of Complications and Flap Loss in Microvascular Reconstructive Surgery: The Role of Fluid Balance, Crystalloid Administration and Operative Time

**DOI:** 10.3390/jcm15145432

**Published:** 2026-07-10

**Authors:** Saeed Torabi, Philipp K. Omuro, Remco Overbeek, Elisabeth H. Adam, Sandra E. Stoll, Tobias Kammerer, Carolin Schroeder, Matthias Zirk, Andrea U. Steinbicker, Fabian Dusse, Max Zinser

**Affiliations:** 1 Department of Anesthesiology and Intensive Care Medicine, Faculty of Medicine, University Hospital of Cologne, University of Cologne, 50931 Cologne, Germany; philipp.omuro@uk-koeln.de (P.K.O.); remco.overbeek@uk-koeln.de (R.O.); sandra.stoll@uk-koeln.de (S.E.S.);; 2 Department of Plastic, Reconstructive and Aesthetic Surgery, Faculty of Medicine, University Hospital of Cologne, University of Cologne, 50931 Cologne, Germany; carolin.schroeder2@gmail.com (C.S.);; 3 Department for Oral and Craniomaxillofacial and Plastic Surgery, Faculty of Medicine, University Hospital of Cologne, University of Cologne, 50931 Cologne, Germany; matthias.zirk@uk-koeln.de

**Keywords:** intraoperative fluid balance, crystalloids, colloids, free-flap surgery, microvascular reconstruction, flap-related complications, goal-directed fluid therapy, head and neck surgery, in-hospital mortality

## Abstract

**Background**: Perioperative fluid therapy plays a critical role in the outcome of microvascular free-flap surgery. While both inadequate and excessive fluid administration may impair flap perfusion and systemic recovery, the impact of fluid balance and crystalloid volume—normalized to body weight and operative time—on postoperative complications remains underexplored. This study investigates the dose-dependent effects of intraoperative fluid and crystalloid administration on flap-related and systemic outcomes. **Methods**: This retrospective, single-centre cohort study included 495 adult patients who underwent microvascular free-flap transplantation between 2009 and 2020. Intraoperative fluid balance and crystalloid volumes were normalized to patient weight and operative duration (mL/kg/h) and stratified into pre-defined thresholds. The primary endpoint was the incidence of flap-related complications (partial/total flap loss, thrombosis, revision surgery). Secondary endpoints included flap loss, suture insufficiency, pneumonia, ICU length of stay (LOS-ICU), and in-hospital mortality. **Results**: Higher intraoperative fluid rates were significantly associated with higher complication rates. Flap-related complications occurred in 54.8% of patients receiving >10 mL/kg/h versus 37.1% in the ≤5 mL/kg/h group (*p* < 0.01) and reached 100% in patients receiving >20 mL/kg/h, although this category comprised only seven patients (*p* < 0.01). Suture insufficiency increased from 3.1% (≤5 mL/kg/h) to 57.1% (>20 mL/kg/h; *p* < 0.01). Pneumonia incidence rose from 8.8% (≤5 mL/kg/h) to 31.9% (>10 mL/kg/h; *p* < 0.01). A U-shaped trend was observed for flap loss, with the highest rate (24.6%) at >10 mL/kg/h. Crystalloid volume > 3000 mL was significantly associated with higher flap loss (20.2% vs. 0.2%; *p* < 0.01) and suture insufficiency (7.0% vs. 0.2%; *p* = 0.02). Red blood-cell (RBC) transfusions were associated with higher overall complication rates (45.6% vs. 34.2%; *p* < 0.01) and suture insufficiency (9.9% vs. 3.4%; *p* < 0.01). Gelatin-based colloids showed no negative impact. Operative time was the only strong independent predictor of total flap loss; each additional operative hour increased the odds of flap loss by 34% (*p* < 0.001). Intraoperative noradrenaline use and a history of neoadjuvant radiotherapy were not independently associated with flap-related complications or flap loss. Median LOS-ICU increased from 2 days to 10 days in patients receiving >20 mL/kg/h (*p* < 0.01). In-hospital mortality increased significantly with higher fluid volumes (0.3% for ≤10 mL/kg/h vs. 28.6% for > 20 mL/kg/h; *p* < 0.01). **Conclusions**: In 495 microvascular free-flap reconstructions, diagnosis, flap type, defect localization and operative time emerged as key determinants of postoperative outcomes, while defect type itself showed no predictive value. Intraoperative fluid overload—particularly crystalloid rates exceeding 10 mL/kg/h—is associated with a significantly higher risk of flap-related complications, pneumonia, prolonged ICU stay and mortality. These findings support the implementation of individualized or goal-directed fluid strategies in microvascular reconstructive surgery to optimize outcomes.

## 1. Introduction

Microvascular free-flap surgery is a cornerstone of reconstructive procedures for head and neck, craniomaxillofacial, extremity, breast and thoracoabdominal defects [1,2]. Despite reported success rates exceeding 90% in high-volume centres, postoperative complications—particularly flap-related events such as partial or total flap loss, wound dehiscence, suture insufficiency and microvascular thrombosis—remain a clinically relevant concern [2,3,4]. Prevention of these complications is critical for preserving flap viability, minimizing the need for revision surgery and optimizing functional and aesthetic outcomes.

Among the numerous intraoperative variables that influence postoperative recovery, fluid management plays a pivotal role [5,6]. Adequate intravascular volume is essential to maintain tissue perfusion across newly created microvascular anastomoses. However, excessive fluid administration may lead to interstitial edema, increased venous pressure and impaired tissue oxygenation, thereby potentially compromising flap integrity [3,6,7]. Conversely, restrictive fluid resuscitation may produce hypoperfusion, thrombotic events and flap failure [8,9]. The optimal fluid strategy must therefore carefully balance these competing risks. In addition, appropriate perioperative anticoagulation management has been identified as another important determinant of microsurgical success [10].

Recent evidence supports the concept of individualized or goal-directed fluid therapy (GDFT) to optimize hemodynamics and reduce complications [11,12,13]. Large meta-analyses of major non-cardiac surgery have demonstrated that GDFT reduces surgical site infections, pulmonary complications and hospital length of stay (LOS) compared with conventional fluid regimens [11,14,15]. In free-flap surgery, prospective and retrospective analyses suggest that liberal fluid administration is associated with higher rates of wound complications, flap revision and prolonged hospitalization [3,5,6,16]. However, current evidence remains limited, and few studies have quantified intraoperative fluid administration using standardized, weight- and time-adjusted indices (e.g., mL/kg/h) that allow interpatient comparability and clinical interpretation. Beyond fluid management, flap loss has been linked to multiple patient-, tumour- and procedure-related factors, including prolonged operative time, recipient-vessel quality and previous neck dissection, venous congestion and microvascular thrombosis, intraoperative hypotension, smoking, diabetes mellitus, peripheral arterial disease and prior radiotherapy [3,4,9]. These determinants frequently coexist and may interact with intraoperative fluid balance.

Historically, liberal intraoperative fluid administration in free-flap surgery has been driven by the intention to preserve flap perfusion and avoid anastomotic thrombosis, by replacement of blood loss during prolonged procedures, and by the limited adoption of goal-directed protocols—factors that may inadvertently result in fluid overload [3,6].

Beyond flap-related outcomes, the broader systemic effects of intraoperative fluid therapy—such as pulmonary complications, prolonged intensive care unit (ICU) stay and in-hospital mortality—have not been comprehensively evaluated in this patient population. Understanding the dose–response relationship between fluid load and these outcomes may help refine perioperative management. A further relevant consideration is fluid composition: emerging data indicate that the choice between crystalloids and balanced or non-balanced colloids may influence postoperative outcomes [17,18,19,20].

To address these knowledge gaps, we conducted a retrospective, single-centre cohort study investigating the association between intraoperative fluid balance—normalized to body weight and operative time—and a range of clinically relevant postoperative outcomes in patients undergoing free-flap reconstruction. The primary endpoint was the incidence of flap-related complications. Secondary endpoints included total flap loss, suture insufficiency, postoperative pneumonia, ICU LOS and in-hospital mortality. We hypothesized that higher intraoperative fluid administration rates would be associated with a stepwise increase in adverse outcomes and that threshold-based analysis might identify clinically relevant inflection points for complication risk.

## 2. Materials and Methods

### 2.1. Study Design and Population

This retrospective, observational, single-centre cohort study analyzed data from patients who underwent microvascular free-flap transplantation at the Department of Oral, Craniomaxillofacial and Plastic Surgery, Faculty of Medicine and University Hospital Cologne, University of Cologne, Germany. All adult patients who underwent elective free-flap transplantation between January 2009 and December 2020 were eligible, irrespective of flap site, donor site, indication and defect localization. After surgery, all patients were transferred to the ICU. Patients were included irrespective of tumour stage (cT1–cT4), nodal status, tumour grading, metastatic status, or whether reconstruction was performed for primary, recurrent, or secondary malignancy. These variables were not used as inclusion or exclusion criteria because the objective of the study was to investigate the association between intraoperative fluid administration and postoperative outcomes following free-flap reconstruction.

Inclusion criteria were:

(1) age ≥ 18 years;
(2) microvascular free-flap reconstruction performed during the study period; and
(3) complete perioperative anesthetic and postoperative outcome data available for analysis.

Exclusion criteria. Patients were excluded if any of the following applied: (i) incomplete intraoperative documentation; (ii) procedures limited to flap revision without new free-flap creation; (iii) intraoperative mortality; (iv) massive transfusion due to surgical bleeding; or (v) administration of coagulation products such as fresh frozen plasma (FFP) or other blood components beyond red blood cells [10]. The study was approved by the Ethics Committee of the Medical Faculty of the University of Cologne (Application No. 24-1305-retro, 15 August 2024); informed consent was waived due to the retrospective nature of the study. Reporting adhered to the STROBE statement for observational studies (Supplementary Materials).

### 2.2. Data Collection

Patient demographics, comorbidities (arterial hypertension, peripheral arterial disease [PAD], chronic obstructive pulmonary disease [COPD], diabetes mellitus, nicotine use) and perioperative variables were extracted from the electronic medical record system (ORBIS, Dedalus HealthCare GmbH, Bonn, Germany). Surgical data included type and duration of the procedure, fluid administration, transfusion requirements and urine output (see Table 1). Postoperative outcomes (complications, ICU LOS, mortality) were obtained from standardized discharge summaries and paper-based ICU documentation. Perioperative antithrombotic and anticoagulation management followed a standardized institutional protocol that has been described and analyzed in detail in a separate study of the same setting [10]; it was therefore not re-evaluated here, the present analysis being confined to intraoperative volume therapy.

### 2.3. Intraoperative Fluid Balance

Intraoperative fluid balance was defined as the total volume of crystalloids, colloids and transfused packed red blood cells (PRBCs) minus blood loss and urine output. To capture the effects of fluid exposure as a dynamic process rather than a static threshold, patients were stratified into pre-defined intraoperative fluid-rate categories. This range-based approach enabled identification of complication trends across increasing levels of fluid administration [1,12].

The intraoperative fluid balance was calculated as:

Fluid balance = (Crystalloids + Colloids + PRBCs) − (Blood loss + Urine output).

Colloidal fluids consisted exclusively of gelatin-based solutions (Gelafundin^®^ 4%, B. Braun, Melsungen, Germany); hydroxyethyl starch was not used [19,21]. The resulting net fluid balance was normalized to each patient’s body weight (kg) and operative time (hours) to express fluid administration as mL/kg/h. All fluid variables were extracted from the electronic anesthesia records. Blood loss was estimated intraoperatively from suction canister volumes (after subtraction of irrigation fluids) and verified using the operative reports documented by the attending surgeons. Urine output was measured continuously via an indwelling urinary catheter and documented in the anesthesia records. Packed red blood-cell (PRBC) transfusions were recorded as administered volumes (mL).

Patients were stratified according to intraoperative fluid administration rate into a reference group (≤5 mL/kg/h) and four cumulative threshold groups (>5, >10, >15, and >20 mL/kg/h). Each threshold group included all patients receiving fluid volumes above the respective cutoff. For each outcome presented in Table 2, the incidence of complications in each threshold group was compared with that in the reference group (≤5 mL/kg/h) using Pearson’s chi-square test or Fisher’s exact test, as appropriate. Accordingly, the reported *p*-values represent comparisons between each cumulative threshold group and the reference group. This stratification was chosen to reflect a range-based exposure model consistent with current ERAS recommendations [1].

Intraoperative fluid therapy was guided at the discretion of the attending anaesthesiologist, based on clinical judgement and continuous invasive arterial pressure monitoring. The primary hemodynamic target was to maintain a mean arterial pressure (MAP) > 65 mmHg. Crystalloids (balanced or 0.9% saline) were the primary volume-replacement fluid, supplemented by norepinephrine as the sole vasopressor when indicated [21,22]. Advanced hemodynamic monitoring parameters such as pulse-pressure variation (PPV) and stroke-volume variation (SVV) were applied inconsistently and not reliably documented; they were therefore excluded from statistical analysis.

Crystalloids were balanced electrolyte solutions (e.g., Sterofundin ISO, B. Braun). The only colloid administered was 4% succinylated gelatin (Gelafundin 4%, B. Braun). Hydroxyethyl starch was not used. Packed red blood cells were recorded in millilitres.

### 2.4. Classification of Defects and Flaps

Defects were categorized according to localization (head and neck, extremities, thoracic/abdominal wall), type (soft tissue, bone, or composite), and flap donor site (upper limb, lower limb, thoracic/abdominal region). Lower-limb flaps included, among others, the anterolateral thigh perforator (ALTP) flap, the fibula flap and the gracilis flap; thoracic/abdominal flaps included the latissimus dorsi, pectoralis major and deep inferior epigastric perforator (DIEP) flaps. This classification ensured consistency with established literature and allowed clinically meaningful subgroup analyses [2,4].

### 2.5. Definition of Flap-Related Complications

Flap-related complications were defined as all clinically relevant postoperative events directly affecting flap viability or requiring flap-specific intervention during the index hospital admission. These included total or partial flap loss, surgical flap revision, arterial or venous thrombosis, revision of the microvascular anastomosis, wound dehiscence, hematoma requiring surgical evacuation, and total or partial skin-graft failure associated with the free flap [2].

Total flap loss was defined as complete necrosis of the transplanted free flap requiring surgical removal, whereas partial flap loss referred to partial tissue necrosis not requiring complete flap removal [2].

All postoperative complications were identified retrospectively from the electronic medical records, operative reports, ICU documentation, and discharge summaries. Complications were recorded only if they occurred during the index hospital admission.

### 2.6. Endpoints

Primary endpoint. Occurrence of flap-related complications, stratified by intraoperative fluid volume normalized to body weight and operative time.

Secondary endpoints. Flap loss in relation to intraoperative fluid and crystalloid volumes; suture insufficiency in relation to intraoperative fluid and transfusion parameters; incidence of postoperative pneumonia; overall postoperative complication rate (including but not limited to flap-related events); impact of red blood-cell transfusion on flap-related complications; ICU length of stay (LOS-ICU); and postoperative in-hospital mortality.

### 2.7. Statistical Analysis

All statistical analyses were performed using IBM SPSS Statistics, version 27.0 (IBM Corp., Armonk, NY, USA). Continuous variables were tested for normality using the Shapiro–Wilk test and are reported as median and interquartile range (IQR). Categorical variables are presented as absolute frequencies and percentages.

Categorical variables (e.g., complication rates, flap loss, pneumonia) were compared using chi-square or Fisher’s exact test, as appropriate. For continuous non-normally distributed outcomes (e.g., ICU LOS), the Kruskal–Wallis or Mann–Whitney U-test was applied. A two-sided *p*-value < 0.05 was considered statistically significant.

Multivariable logistic regression analyses were performed to identify independent predictors of flap-related complications and total flap loss. Variables entered into the model were age, sex, operative time, crystalloid volume, intraoperative urine output, defect characteristics and indication. Results are reported as odds ratios (OR) with 95% confidence intervals (CI) and corresponding *p*-values.

## 3. Results

A total of 495 patients who underwent microvascular free-flap surgery were included. The overall flap-related complication rate was 38.0% (n = 188); the total flap-loss rate was 17.0% (n = 84); the suture-insufficiency rate was 5.7% (n = 28).

### 3.1. Patient Characteristics

Baseline demographic characteristics did not differ significantly between patients with and without flap-related complications, with the exception of peripheral arterial disease (PAD), which was significantly more prevalent among patients who developed flap-related complications (9.0% vs. 4.0%; *p* = 0.02). Age, sex, hypertension, COPD, diabetes and nicotine use showed no significant association with complication occurrence (Table 1).

### 3.2. Impact of Diagnosis, Flap Type and Defect Characteristics

Indications were grouped into four categories: tumour (n = 427), infection (n = 25), trauma (n = 19) and other (n = 24). Tumour-related reconstructions represented the vast majority of cases and showed the lowest complication rate (34%) and flap-loss rate (14%). Infection-related reconstructions and “other” indications showed the highest complication rates (60% and 58%, respectively) and the highest flap-loss rates (28% and 33%; *p* < 0.05 for trend).

Flap type was categorized as: (1) upper-limb flaps (radial forearm flap, n = 244); (2) lower-limb flaps (ALTP and fibula flaps, n = 174); and (3) thoracic/abdominal-wall flaps (latissimus dorsi and pectoralis major flaps, n = 77). Complication rates were 35%, 46% and 30%, respectively; flap-loss rates were 15%, 21% and 14% (*p* < 0.05 for lower-limb vs. other groups).

Defect localization was classified as: (1) intraoral; (2) head/neck extraoral; (3) extremity (arm/leg); and (4) thoracic/abdominal wall. Complication rates ranged from 18% (thoracic/abdominal wall) to 58% (head/neck extraoral). Pairwise comparisons showed significantly higher complication rates for head/neck extraoral and extremity defects than for thoracic/abdominal-wall defects (*p* < 0.01).

Defect type was categorized as soft tissue only, bone only or composite. Complication rates ranged between 34% and 40%, with flap-loss rates of approximately 17% in all groups. No significant association was identified between defect type and either endpoint (*p* = 0.95 for complications; *p* = 0.69 for flap loss).

In summary, diagnosis, flap type and defect localization were significant predictors of postoperative complications and flap loss. Tumour cases had the most favourable outcomes, whereas trauma carried the highest risk. Extremity defects and fibula flaps were particularly associated with higher rates of complications and flap loss.

### 3.3. Neoadjuvant Radiotherapy in Tumour Patients

Among the 427 patients undergoing free-flap reconstruction for tumour-related defects, 55 (12.9%) had a history of prior radiotherapy. Flap-related complications occurred in 38% (21/55) of irradiated patients versus 33% (124/372) of non-irradiated patients (Fisher’s exact *p* = 0.54). Total flap loss was observed in 20% (11/55) of irradiated patients compared with 14% (50/372) of non-irradiated patients (*p* = 0.22). In multivariable logistic regression adjusting for age and operative time, prior radiotherapy was not independently associated with complications (OR 1.10; *p* = 0.75) or flap loss (OR 1.27; *p* = 0.54).

### 3.4. Impact of Intraoperative Fluid Volume on Flap-Related Outcomes

Increasing intraoperative fluid administration, normalized to body weight and operative time, was significantly associated with higher rates of flap-related complications. Patients receiving >10 mL/kg/h had a markedly higher complication rate than those receiving ≤5 mL/kg/h (54.8% vs. 37.1%; *p* < 0.01). This association intensified at higher thresholds, reaching 71.4% for >15 mL/kg/h and 100% for >20 mL/kg/h (*p* < 0.01 for both).

Suture-insufficiency rates increased progressively with fluid volume: 3.1% (≤5 mL/kg/h), 17.1% (>10 mL/kg/h), 33.3% (>15 mL/kg/h) and 57.1% (>20 mL/kg/h); all associations were statistically significant (*p* < 0.01).

Pneumonia incidence rose significantly with increasing fluid load: 8.8% (≤5 mL/kg/h), 18.7% (>5 mL/kg/h; *p* < 0.01) and 31.9% (>10 mL/kg/h; *p* < 0.01).

Although flap-loss rates numerically increased with higher fluid administration (e.g., 14.9% for ≤5 mL/kg/h vs. 24.6% for >10 mL/kg/h), this trend did not reach statistical significance in all comparisons (Table 2).

These highest categories comprised very few patients (n = 21 for >15 mL/kg/h and n = 7 for >20 mL/kg/h); the resulting estimates, including the 100% complication rate at >20 mL/kg/h, are based on small denominators with wide confidence intervals and must be interpreted with caution.

### 3.5. Intraoperative Fluid Therapy

The median perioperative fluid balance was +3150 mL [IQR 2050–4900]. The mean intraoperative fluid administration consisted of crystalloids 5000 mL [IQR 3500–7000], colloids 0 mL [IQR 0–500] and red blood-cell transfusions, which were administered in 37.8% (n = 187) of cases. The median operative time was 480 min [IQR 392–578].

### 3.6. Crystalloid Volume and Outcomes

Crystalloid administration > 1000 mL was significantly associated with increased complication rates (38.6% vs. 24.4% for ≤ 1000 mL; *p* = 0.04) and a higher incidence of flap loss (18.3% vs. 0.2%; *p* < 0.01). This association remained significant at thresholds of >3000 mL and > 4000 mL for flap loss (*p* < 0.01). Crystalloid volume > 3000 mL was also associated with a significantly higher rate of suture insufficiency (7.0% vs. 0.2%; *p* = 0.02) (Table 3).

### 3.7. Colloids and Transfusions

In 47.8% (n = 160) of cases, colloidal fluids (gelatin) were administered perioperatively. Patients receiving colloids showed no increase in flap-related complications (38.1% vs. 37.9%; χ^2^ = 0.002), total flap loss (17.6% vs. 16.7%; χ^2^ = 0.061), suture insufficiency (8.1% vs. 4.5%; χ^2^ = 2.699) or in-hospital mortality (1.3% vs. 1.0%; χ^2^ = 0.003).

Patients who received red blood-cell (RBC) transfusions experienced significantly higher rates of overall complications (45.6% (171) vs. 34.2% (110); χ^2^ = 6.209; *p* < 0.01) and suture insufficiency (9.9% (17) vs. 3.4% (11); χ^2^ = 8.878; *p* < 0.01) compared with non-transfused patients. In-hospital mortality (2.3% (4) vs. 0.6% (2); χ^2^ = 2.742) and total flap loss (20.0% (34) vs. 15.5% (50); χ^2^ = 1.572) did not differ significantly between groups.

### 3.8. Operative Duration

Operative time ranged from 69 min to over 17 h, with a median of 480 min (8 h). Logistic regression analysis identified operative time as a highly significant independent predictor of flap loss (*p* = 2.5 × 10^−8^). The odds ratio was 1.0047 per minute (95% CI 1.0030–1.0064), corresponding to a 34% increase in odds of flap loss per additional operative hour. The probability of flap loss increased from 3% at 4 h to 9% at 8 h and >20% beyond 12 h, holding all other variables at median values.

### 3.9. Vasopressor Use and Outcomes

Of the 495 patients with complete ICU records, 160 (32%) received noradrenaline upon ICU admission and during the first ICU shift, with a median dose of 0.10 µg/kg/min (IQR 0.06–0.22). Patients receiving noradrenaline had a flap-related complication rate of 44% compared with 35% in those without vasopressor support (*p* = 0.05). After multivariable adjustment (age, sex, operative time, crystalloid volume), noradrenaline use was not independently associated with flap-related complications (OR 1.18; 95% CI 0.78–1.78; *p* = 0.44).

For total flap loss, raw rates were 21% with noradrenaline versus 15% without (*p* = 0.18). After adjustment, noradrenaline use was not independently associated with flap loss (OR 1.26; 95% CI 0.75–2.10; *p* = 0.37), while operative time remained a strong independent predictor (OR 1.005 per minute; *p* < 0.001).

### 3.10. Multivariable Analysis of Flap-Related Complications and Flap Loss

A total of 459 cases had complete data on age, sex, operative time, crystalloid volume, intraoperative urine output, flap-related complications and flap loss, and were included in the multivariable analysis. Median age was 63 years (IQR 54–72), and 31% of patients were female. Median operative time was 480 min (IQR 392–578). Patients received a median of 5.0 L crystalloids (IQR 3.5–7.0), resulting in a net fluid balance of 3.3 L (IQR 2.1–5.0).

In multivariable logistic regression for any flap-related complication, none of the included variables reached statistical significance. Operative time showed a non-significant upward trend (OR 1.001 per minute; 95% CI 0.999–1.002; *p* = 0.12).

When total flap loss was used as the dependent variable, operative time emerged as a strong, independent risk factor (OR 1.004 per minute; 95% CI 1.002–1.006; *p* < 0.001), corresponding to a 34% increase in the odds per additional operative hour. Crystalloid volume showed a marginal association (OR 1.19 per litre; 95% CI 0.98–1.45; *p* = 0.076). Age, sex and net fluid balance were not independently related to flap loss. Adjusted probability calculations demonstrated a stepwise increase in risk with longer procedures: 3% at 4 h, 9% at 8 h and >20% beyond 12 h. Sensitivity analyses confirmed this association when operative time was categorized (<4 h, 4–8 h, >8 h; *p*-trend < 0.001).

### 3.11. Mortality and ICU Length of Stay

In-hospital mortality increased significantly with higher intraoperative fluid administration. Mortality was 5.7% (4/70) in patients receiving >10 mL/kg/h compared with 0.3% (1/425) in those receiving ≤10 mL/kg/h (χ^2^ = 16.672; *p* < 0.01); 9.5% (2/21) for >15 mL/kg/h compared with 0.7% (3/474) in those receiving ≤ 15 mL/kg/h (χ^2^ = 14.758; *p* < 0.01); and 28.6% (2/7) for >20 mL/kg/h compared with 0.7% (3/488) in those receiving ≤20 mL/kg/h (*p* < 0.01).

**Figure 1:**
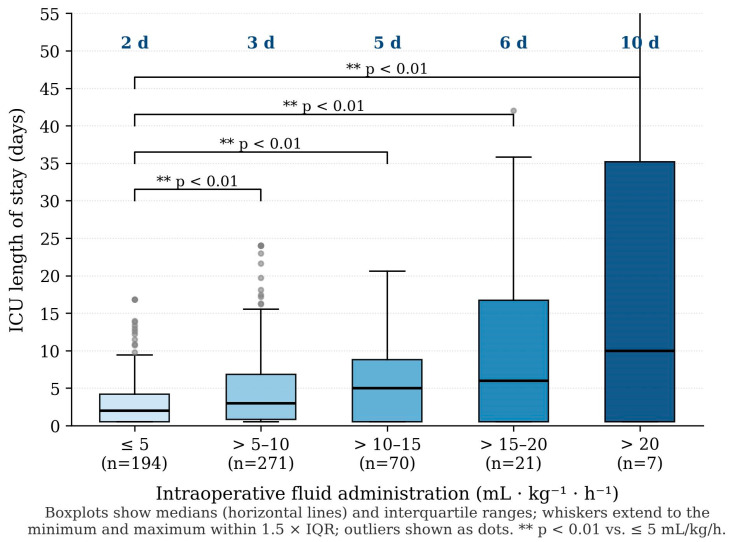
Length of stay in the intensive care unit (LOS-ICU) according to intraoperative fluid administration normalized to body weight (kg) and operative time (h). Boxplots show medians and interquartile ranges; whiskers indicate minimum and maximum values. Between-group differences were tested with the Kruskal–Wallis test (overall) and the Mann–Whitney U-test (pairwise versus ≤5 mL/kg/h). mL, millilitre; kg, kilogram; h, hour.

Median LOS-ICU for the overall cohort was 2 days [IQR 1–5]. When stratified by fluid therapy, LOS-ICU differed significantly between groups: >5 mL/kg/h (3 (1–7) days; *p* < 0.01), >10 mL/kg/h (5 (2–11) days; *p* < 0.01), > 15 mL/kg/h (6 (2–21) days; *p* < 0.01) and >20 mL/kg/h (10 (6–42) days; *p* < 0.01) (Figure 1).

### 3.12. Summary of Results

**Figure 2:**
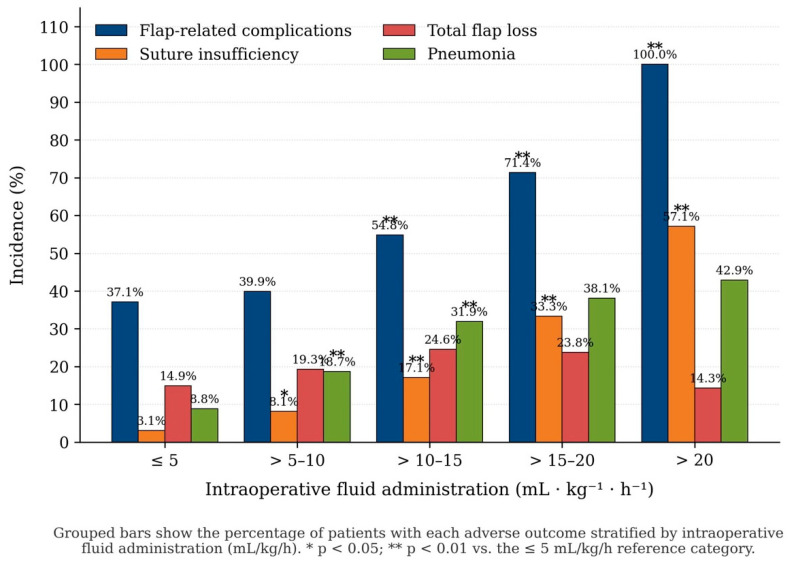
Postoperative complications by intraoperative fluid rate (mL/kg/h). Bars show the percentage of patients in each fluid stratum experiencing flap-related complications, suture insufficiency and pneumonia. Denominators per stratum are as in Table 2; the highest strata (>15 and >20 mL/kg/h) contain few patients (n = 21 and n = 7), so the corresponding proportions should be interpreted with caution. Complications [%] are shown by fluid administration normalized to body weight (kg) and operative time (h).

The graphical representation demonstrates a clear and consistent association between higher intraoperative fluid administration and an increased incidence of postoperative complications in microvascular free-flap surgery (Figure 2).

Flap-related complication rates increased progressively with fluid volume: from 37.1% in patients receiving ≤5 mL/kg/h to 54.8% at >10 mL/kg/h, 71.4% at >15 mL/kg/h and 100% at >20 mL/kg/h. This pattern indicates a strong and dose-dependent relationship between fluid overload and the risk of complications.

Suture insufficiency followed a similar pattern, increasing steadily from 3.1% in the low-volume group to 17.1% at >10 mL/kg/h and peaking at 57.1% in patients who received >20 mL/kg/h. These findings suggest a substantial impact of fluid volume on anastomotic integrity and wound healing.

Pneumonia incidence also increased with higher fluid volumes, rising from 8.8% in the lowest fluid group to 31.9% at >10 mL/kg/h. This may reflect pulmonary compromise related to fluid overload and impaired respiratory function. In contrast, flap loss showed a more moderate and inconsistent increase, ranging from 14.9% (≤5 mL/kg/h) to 24.6% at >10 mL/kg/h, suggesting a U-shaped rather than purely linear relationship.

## 4. Discussion

In this single-centre retrospective cohort study of 495 patients undergoing microvascular free-flap reconstruction, we identified a clear dose-dependent association between intraoperative fluid administration—particularly crystalloid volume normalized to body weight and operative time—and a wide spectrum of postoperative outcomes. Fluid rates exceeding 10 mL/kg/h were associated with significantly higher incidences of flap-related complications, suture insufficiency and pneumonia. Flap loss demonstrated a U-shaped relationship, suggesting that both hypovolemia and hypervolemia adversely affect flap viability. Higher fluid exposures were further linked to prolonged ICU stay and increased in-hospital mortality.

Our finding that flap-related complications and suture insufficiency increase markedly with higher crystalloid infusion rates is consistent with prior evidence in reconstructive surgery. Burkhard et al. [5] identified a number of perioperative predictors of early surgical revision and flap-related complications, including intraoperative volume management. Dooley et al. [6] reported that both intraoperative and post-anesthesia-care-unit fluid administration acted as independent risk factors for postoperative complications in patients undergoing free tissue transfer for head and neck cancer. Pattani et al. [3] further demonstrated in a comprehensive review of the literature that liberal intraoperative fluid administration constitutes one of the principal modifiable risk factors for flap failure. Booi [7] specifically showed that perioperative fluid overload increased anastomotic thrombosis in free TRAM flaps, and Rhee et al. [8] confirmed that a fluid-restrictive strategy is associated with fewer flap-related complications. This bidirectional risk underscores that postoperative antithrombotic management [10] and intraoperative fluid stewardship are interdependent determinants of flap viability and surgical success.

Recent evidence highlights a growing trend toward goal-directed and individualized fluid management in perioperative care, particularly in microvascular reconstruction. Tapia et al. [23] demonstrated that implementation of goal-directed fluid therapy (GDFT) in patients undergoing head and neck free-flap procedures significantly reduced flap necrosis rates from 37.1% to 13.6%, shortened ICU stays and improved overall surgical outcomes compared with conventional management. Lahtinen et al. [24] reported analogous benefits of GDFT in head and neck free-flap cohorts, with reductions in net fluid administration and no compromise in flap outcomes. These findings are corroborated by the foundational work of Gan et al. [11] and Meng and Heerdt [12], which established that flow-based GDFT shortens hospital stay and improves postoperative recovery after major non-cardiac surgery, and by the GDFT meta-analyses by Chong et al. [14] and Sun et al. [15], which collectively demonstrate lower rates of pulmonary complications, surgical site infections and shorter hospital stays. A systematic review of perioperative fluid management in free-flap reconstructive surgery by Shah et al. [16] similarly concluded that liberal fluid regimens were consistently associated with adverse flap outcomes. In contrast, Zheng et al. [25] reported that within their cohort, intraoperative fluid management exerted limited influence on surgical outcomes—an observation that may reflect differences in baseline fluid practices and underscores the importance of context-specific interpretation of volume thresholds.

The association between suture insufficiency and excessive crystalloid load reflects fundamental physiological principles. Interstitial edema from crystalloid overload increases tissue pressure, compromises anastomotic microcirculation and impairs wound healing. Recent mechanistic evidence by Sukudom et al. [26] has demonstrated a clear association between intravenous fluid administration and endothelial glycocalyx shedding, providing a molecular substrate for fluid-induced microvascular injury. Few previous studies have differentiated the impact of crystalloids from total fluid volume. Our data show that crystalloid volume alone, particularly when exceeding 3000 mL or 10 mL/kg/h, is significantly associated with adverse microsurgical outcomes—including higher rates of flap-related complications, suture insufficiency and flap loss. These findings reinforce the growing consensus that both the quantity and the composition of intraoperative fluid administration are critical determinants of microsurgical success [17,18,19,20,27,28]. Excessive crystalloid use, even independent of total fluid balance, may adversely affect tissue perfusion and compromise flap viability.

In our cohort, the intraoperative use of noradrenaline at a median rate of 0.10 µg/kg/min was not associated with an increased risk of flap-related complications or flap loss. This finding is consistent with multiple prospective and retrospective series demonstrating that judicious vasopressor use does not compromise free-flap perfusion and may instead provide a useful tool to maintain MAP without further fluid escalation [21,22,29]. The systematic review by Naik et al. [21] and the analyses by Fang et al. [22] and Munro et al. [29] collectively demonstrate that intraoperative vasopressor administration does not increase the risk of flap compromise in cancer patients undergoing free tissue transfer. These data support the modern paradigm of demand-adapted vasopressor support to maintain organ perfusion while limiting fluid overload.

Intraoperative red blood-cell (RBC) transfusions were associated with a higher rate of overall postoperative complications (45.6% vs. 34.2%) and a markedly increased incidence of suture insufficiency (9.9% vs. 3.4%) in our cohort. These results are in line with the systematic review and meta-analysis by Giovacchini et al. [30], which demonstrated a significant association between perioperative blood transfusions and complications in head and neck reconstruction. By contrast, colloid administration—exclusively gelatin-based solutions in our cohort—was not associated with adverse outcomes. This finding aligns with the randomized trial by László et al. [18], which compared crystalloids with 6% hydroxyethyl starch (HES) in free-flap surgery and showed that achieving hemodynamic targets required approximately 1.5 times more crystalloid than colloid, with no measurable benefit in microcirculatory perfusion. The interpretation of these findings has been the subject of an editorial exchange [17,19,28], emphasizing the ongoing controversy regarding the optimal composition of GDFT solutions. The use of HES in this context has been widely restricted because of known renal and coagulopathic effects [21]; in our cohort, colloid therapy consisted exclusively of gelatin-based solutions, and no association with increased postoperative complications was observed, suggesting that judicious use of gelatin may serve as a volume-efficient alternative to excessive crystalloid infusion. A recent 2024 meta-analysis by Niu et al. [20], including 15 randomized controlled trials and 2956 non-cardiac surgical patients, found no overall advantage of colloids over crystalloids when administered as part of a GDFT protocol, although colloids were associated with fewer gastrointestinal complications.

In our cohort, pneumonia rates increased steeply from 8.8% in lower fluid categories to over 30% at >10 mL/kg/h. This finding aligns with broader perioperative evidence that positive fluid balance contributes to pulmonary edema and infection. The clinical review by Voldby and Brandstrup [31] emphasized that zero-balance fluid therapy in non-cardiac surgery significantly reduces postoperative complications, particularly respiratory events; Benes et al. [32] similarly highlighted the dual-edged nature of fluid therapy in critical care, with both insufficient and excessive volumes contributing to morbidity. Together with the GDFT meta-analyses [14,15], these data support a more restrictive or zero-balance strategy as the default in major surgery, complemented by individualized GDFT where feasible.

The U-shaped pattern observed in flap loss, although not always statistically significant, suggests that both extremes of intraoperative fluid therapy may adversely affect flap viability. Hypovolemia and intraoperative hypotension can lead to inadequate tissue perfusion and increase thrombotic risk [9], whereas hypervolemia may cause interstitial edema, venous congestion and compromised microcirculation [3,7]. These findings reinforce the rationale for a balanced or goal-directed fluid strategy that avoids both fluid overload and under-resuscitation. In line with this, the Enhanced Recovery After Surgery (ERAS) Society recommends an individualized goal-directed approach for major head and neck free-flap surgery [1].

Similar to the recent observations of Ghaffar et al. [33], our study shows that higher intraoperative fluid rates are associated with prolonged ICU stays after free-flap surgery. Analogous observations from general surgery indicate that fluid volume quintiles above approximately 16–26 mL/kg/h significantly increase odds of prolonged hospitalization and complications [14,15]. Our findings further demonstrate a clear dose-dependent relationship between intraoperative fluid administration and both in-hospital mortality and ICU LOS. Although overall mortality was low, the increased rates observed in patients receiving higher fluid volumes likely reflect a cumulative effect of factors such as flap failure, infectious complications and prolonged intensive care, suggesting that liberal fluid therapy may significantly contribute to postoperative morbidity and mortality.

Operative time emerged as a strong, independent predictor of flap loss in our multivariable analysis. Each additional operative hour was associated with a 34% increase in the odds of flap loss, with the probability rising from 3% at 4 h to over 20% beyond 12 h. These findings are consistent with previous large-cohort analyses and the systematic review by Walia et al. [4], which identified prolonged operative duration as one of the most robust determinants of adverse outcomes after head and neck free-flap reconstruction.

In multivariable analysis, operative time—rather than any fluid variable—remained the only independent predictor of total flap loss. The univariable associations between higher fluid rates and adverse outcomes may therefore be partly mediated or confounded by procedure duration and case complexity, and should be interpreted as hypothesis-generating rather than causal.

Flap outcomes differed markedly by anatomical region. Complication rates ranged from 18% for thoracic/abdominal-wall defects to 58% for extraoral head-and-neck defects, and head-and-neck and extremity defects had significantly higher complication rates than thoracic/abdominal-wall defects (*p* < 0.01); lower-limb flaps (including fibula) showed the highest flap-loss rate (21%). These regional differences are plausibly explained by factors like frequent prior irradiation of head-and-neck fields, by constrained recipient-vessel options and longer composite reconstructions, and by the dependent position and atherosclerotic burden relevant to extremity flaps. Importantly, anatomical region and flap type were accounted for in the multivariable analysis, in which operative time remained the dominant independent predictor of total flap loss.

The strong, independent association underscores the clinical importance of surgical efficiency, multidisciplinary team coordination and strategies aimed at minimizing operative duration—e.g., two-team approaches, parallel donor-site and recipient-site dissection, and standardized perioperative pathways—as central tenets of complication prevention [1,5].

As an additional finding, our cohort analysis showed that preoperative neoadjuvant radiotherapy had no adverse effect on flap survival or flap-related complications after adjustment for age and operative time. This contrasts with the recent meta-analysis by Miller et al. [34], which reported an increased risk of total complications, flap failure and fistula formation in irradiated patients undergoing head and neck reconstruction. The discrepancy may reflect differences in patient selection, surgical timing relative to radiotherapy, or the proportion of high-risk reconstructions in the present cohort.

The total flap-loss rate of 17% in our cohort exceeds the numbers reported by some high-volume centres. This most likely reflects the case-mix of a tertiary referral centre, including a high proportion of complex composite and osseous reconstructions, fibula and extremity flaps, and trauma-, infection- and salvage-related indications, together with the broad study period that encompasses changing surgical and anesthetic practice and an institutional learning curve

### 4.1. Clinical Implications

Our findings translate into several practical implications for perioperative care in microvascular reconstructive surgery: (i) target intraoperative net fluid rates ≤ 10 mL/kg/h, with vigilant monitoring [6,8]; (ii) implement GDFT protocols when feasible, using dynamic parameters such as SVV/PPV [12,15]; (iii) employ noradrenaline early to maintain MAP > 65 mmHg rather than escalating crystalloid administration [21,22]; (iv) monitor the net crystalloid balance perioperatively to minimize pulmonary risk and shorten ICU stay [31,32]; and (v) recognize surgical efficiency and strategies to minimize operative duration as essential elements to optimize flap survival [4]. Preoperative neoadjuvant radiotherapy, in our cohort, was not associated with increased rates of flap-related complications or flap loss after multivariable adjustment, although this finding warrants further confirmation in prospective studies [34].

### 4.2. Limitations

Our study is limited by its retrospective, single-centre design and the lack of standardized recording of dynamic hemodynamic parameters such as PPV and SVV. Complication and pneumonia ascertainment relied on chart review and may be subject to under-reporting. The wide time span (2009–2020) may also reflect evolving anesthetic and surgical practice patterns. Surgical technique, surgeon experience and patient complexity may confound the observed associations. Although multivariable logistic regression was performed for the principal outcomes, residual confounding cannot be excluded, and the threshold-based stratification approach—while clinically intuitive—does not capture the full continuous dose–response relationship. Finally, the relatively small number of patients in the highest fluid-rate categories (>15 and >20 mL/kg/h) limits the precision of effect estimates at these extremes.

Detailed oncological data (TNM stage, histological grade and subtype) were not systematically available across the study period and were not included; outcomes were therefore not stratified by tumour stage.

### 4.3. Future Directions

Prospective, multi-centre studies are needed to validate fluid thresholds and individualized GDFT protocols specifically in reconstructive microsurgery. Future designs should incorporate dynamic perfusion monitoring (e.g., near-infrared spectroscopy or infrared fluorescent angiography) and prospectively define standardized outcomes [2,4]. Longitudinal follow-up could clarify how intraoperative fluid choices affect long-term morbidity, flap viability and quality of life.

## 5. Conclusions

In this large cohort of 495 free-flap reconstructions, diagnosis, flap type, defect localization, and operative time emerged as key determinants of postoperative outcomes, while defect type itself showed no predictive value. Tumour-related cases had the most favourable outcomes, whereas trauma, infection and other indications carried the highest risk for both complications and flap loss. Extremity defects and fibula flaps were particularly associated with elevated flap-failure rates. Prolonged operative duration was a strong, independent predictor of flap loss, with each additional operative hour increasing the risk by approximately one-third [4]. Among tumour patients, a history of prior neoadjuvant radiotherapy was not associated with increased rates of flap-related complications or flap loss after adjustment for age and operative time [34].

Intraoperative fluid overload—particularly crystalloid volumes exceeding 10 mL/kg/h—is associated with a significantly higher risk of flap-related complications, suture insufficiency, pneumonia, prolonged ICU stay and in-hospital mortality. Higher fluid administration may partly reflect longer or more complex procedures. These findings support the implementation of individualized, goal-directed fluid strategies in microvascular reconstructive surgery [1,15,23], complemented by early use of vasopressors to maintain target perfusion pressures [21,22], in order to optimize patient outcomes.

## Figures and Tables

**Table 1:** Baseline characteristics of the study population. Demographics for all patients undergoing microvascular free-flap surgery are shown, as well as subgroup comparisons between patients with and without flap-related complications. Data are presented as percentages [%] and absolute numbers [n] or as median [IQR], as appropriate. PAD, peripheral arterial disease; COPD, chronic obstructive pulmonary disease; n.s., not significant.

Item	Overall	With Complications % [n]	Without Complications % [n]	*p*-Value
Sex, % [n]	M 54.1% [268]/F 45.9% [227]	M 54.8% [103]/F 45.2% [85]	M 53.7% [165]/F 46.3% [142]	n.s.
Age [years], median [IQR]	62 [53–72]	62 [53–71]	62 [53–73]	n.s.
Arterial hypertension, % [n]	40.3% [198]	39.4% [74]	41.1% [124]	n.s.
PAD, % [n]	5.9% [29]	9.0% [17]	4.0% [12]	0.02
COPD, % [n]	14.4% [71]	16.0% [30]	13.5% [41]	n.s.
Nicotine use, % [n]	20.3% [100]	19.1% [36]	21.1% [64]	n.s.
Diabetes mellitus, % [n]	13.8% [68]	14.9% [28]	13.2% [40]	n.s.

**Table 2:** Impact of intraoperative fluid volume on flap-related complications, suture insufficiency, flap loss and pneumonia. Data are presented as percentages [%] and absolute numbers [n]. Intraoperative fluid volume was normalized to body weight and operative time (mL/kg/h) and categorized into clinically relevant strata.

Fluid Category	Overall % [n]	Event % [n]	No Event % [n]	*p*-Value
Flap-related complications				
Overall	100% [495]	38.0% [188]	62.0% [307]	—
≤5 mL/kg/h	39.2% [194]	37.1% [72]	62.9% [122]	—
>5 mL/kg/h	54.7% [271]	39.9% [108]	60.2% [163]	n.s.
>10 mL/kg/h	14.1% [70]	54.8% [38]	45.7% [32]	<0.01
>15 mL/kg/h	4.2% [21]	71.4% [15]	28.6% [6]	<0.01
>20 mL/kg/h	1.4% [7]	100% [7]	0% [0]	<0.01
Total flap loss				
Overall	100% [495]	17.0% [84]	82.8% [410]	—
≤5 mL/kg/h		14.9% [29]	85.1% [165]	—
>5 mL/kg/h		19.3% [52]	80.7% [218]	n.s.
>10 mL/kg/h		24.6% [17]	75.4% [52]	n.s.
>15 mL/kg/h		23.8% [5]	76.2% [16]	n.s.
>20 mL/kg/h		14.3% [1]	85.7% [6]	n.s.
Suture insufficiency				
Overall	100% [495]	5.7% [28]	94.3% [467]	—
≤5 mL/kg/h		3.1% [6]	96.9% [188]	—
>5 mL/kg/h		8.1% [22]	91.9% [249]	0.02
>10 mL/kg/h		17.1% [12]	82.9% [58]	<0.01
>15 mL/kg/h		33.3% [7]	66.7% [14]	<0.01
>20 mL/kg/h		57.1% [4]	42.9% [3]	<0.01
Pneumonia				
≤5 mL/kg/h		8.8% [17]	91.2% [176]	—
>5 mL/kg/h		18.7% [50]	81.3% [217]	<0.01
>10 mL/kg/h		31.9% [22]	68.1% [47]	<0.01
>15 mL/kg/h		38.1% [8]	61.9% [13]	n.s.
>20 mL/kg/h		42.9% [3]	57.1% [4]	n.s.

**Table 3:** Impact of intraoperative crystalloid volume on flap-related complications, total flap loss and suture insufficiency. Intraoperative crystalloid volume was categorized into clinically relevant thresholds and analyzed for its association with adverse outcomes.

Crystalloid Category	Overall % [n]	Event % [n]	No Event % [n]	*p*-Value
Flap-related complications				
≤1000 mL	8.3% [41]	24.4% [10]	75.6% [31]	—
>1000 mL	89.7% [444]	38.6% [170]	61.4% [270]	0.04
>2000 mL	85.9% [425]	39.3% [167]	60.7% [258]	n.s.
>3000 mL	74.3% [368]	40.5% [149]	59.5% [219]	0.03
>4000 mL	61.2% [303]	39.3% [119]	60.7% [184]	n.s.
Total flap loss				
≤1000 mL		2.4% [1]	97.6% [40]	—
>1000 mL		18.3% [81]	81.7% [362]	<0.01
>2000 mL		18.4% [78]	81.6% [346]	0.10
>3000 mL		20.2% [74]	79.8% [293]	<0.01
>4000 mL		20.5% [62]	79.5% [240]	<0.01
Suture insufficiency				
≤1000 mL		0.2% [1]	99.8% [40]	—
>1000 mL		6.1% [27]	93.9% [417]	n.s.
>2000 mL		6.4% [27]	93.6% [398]	n.s.
>3000 mL		7.0% [26]	93.0% [342]	0.02
>4000 mL		7.3% [22]	92.7% [281]	n.s.

## Data Availability

The datasets generated and analyzed during the current study are available from the corresponding author upon reasonable request.

## Supplementary Materials

The following supporting information can be downloaded at: https://www.mdpi.com/article/10.3390/jcm15145432/s1, STROBE Statement Checklist. Refs. [35,36] are cited in Supplementary Materials.

## Abbreviations

ALTP, anterolateral thigh perforator; CI, confidence interval; COPD, chronic obstructive pulmonary disease; DIEP, deep inferior epigastric perforator; ERAS, Enhanced Recovery After Surgery; FFP, fresh frozen plasma; GDFT, goal-directed fluid therapy; HES, hydroxyethyl starch; ICU, intensive care unit; IQR, interquartile range; LOS, length of stay; MAP, mean arterial pressure; NIRS, near-infrared spectroscopy; OR, odds ratio; PAD, peripheral arterial disease; PPV, pulse-pressure variation; RBC, red blood cell; STROBE, Strengthening the Reporting of Observational Studies in Epidemiology; SVV, stroke-volume variation.
