# Supplementary material for: Perioperative Predictors of Complications and Flap Loss in Microvascular Reconstructive Surgery: The Role of Fluid Balance, Crystalloid Administration and Operative Time
Source: J Clin Med. 2026 Jul 10;15(14):5432. doi: 10.3390/jcm15145432 (PMC13411073; doi:10.3390/jcm15145432)
Supplement: Supplementary file 1 [file jcm-15-05432-s001.zip › jcm-4355827-supplementary.pdf]

Supplementary Material — STROBE Statement Checklist

Strengthening the Reporting of Observational Studies in Epidemiology — Cohort Study Checklist (22 items)

Manuscript: Perioperative Predictors of Complications and Flap Loss in Microvascular Reconstructive Surgery: The Role of Fluid Balance, Crystalloid Administration and Operative Time

Corresponding author: Saeed Torabi, MD (saeed.torabi@uk-koeln.de)

Target journal: Journal of Clinical Medicine (MDPI) — Special Issue "Anesthesia in Head and Neck Surgery"

Study design: Retrospective, single-centre cohort study (n = 495)

Reference: *von Elm E, Altman DG, Egger M, Pocock SJ, Gøtzsche PC, Vandenbroucke JP; STROBE Initiative. The Strengthening the Reporting of Observational Studies in Epidemiology (STROBE) statement: guidelines for reporting observational studies. Lancet. 2007;370(9596):1453-7. https://www.strobe-statement.org*

Item No.	Topic	Recommendation (STROBE)	Reported on (manuscript location)
**Title and Abstract**			
1	**Title and abstract**	(a) Indicate the study's design with a commonly used term in the title or the abstract. (b) Provide in the abstract an informative and balanced summary of what was done and what was found.	Title: "Perioperative Predictors of Complications and Flap Loss in Microvascular Reconstructive Surgery…" — retrospective cohort design implicit by content. Abstract (Background, Methods, Results, Conclusions) — p. 1–2; structured summary including n = 495, primary and secondary endpoints, key effect estimates and p-values.
**Introduction**			
2	**Background / rationale**	Explain the scientific background and rationale for the investigation being reported.	Section 1 (Introduction), paragraphs 1–4 — p. 2–3. Discusses clinical importance of flap-related complications, pivotal role of intraoperative fluid management, current evidence gap for weight- and time-adjusted dosing.
3	**Objectives**	State specific objectives, including any pre-specified hypotheses.	Section 1 (Introduction), paragraph 5 — p. 3. Pre-specified hypothesis: higher intraoperative fluid administration rates would be associated with a stepwise increase in adverse outcomes; threshold-based analysis to identify clinically relevant inflection points. Primary and secondary endpoints explicitly listed.
**Methods**			
4	**Study design**	Present key elements of study design early in the paper.	Section 2.1 (Study Design and Population) — p. 3. Explicitly stated: "This retrospective, observational, single-centre cohort study analysed data from patients who underwent microvascular free-flap transplantation."

Item No.	Topic	Recommendation (STROBE)	Reported on (manuscript location)
5	**Setting**	Describe the setting, locations, and relevant dates, including periods of recruitment, exposure, follow-up, and data collection.	Section 2.1 — p. 3. Setting: Department of Oral, Craniomaxillofacial and Plastic Surgery, University Hospital Cologne, Germany. Period: January 2009 – December 2020. Follow-up: until hospital discharge or in-hospital mortality. Postoperative care: ICU.
6	**Participants**	(a) Cohort study — Give the eligibility criteria, and the sources and methods of selection of participants. Describe methods of follow-up. (b) For matched studies, give matching criteria and number of exposed/unexposed.	(a) Section 2.1 — p. 3. Eligibility: all adult (≥ 18 y) patients undergoing free-flap transplantation 2009–2020 irrespective of flap site, donor site, indication or defect localization. Exclusion criteria: incomplete documentation, flap revision only, intraoperative mortality, massive transfusion, FFP/coagulation-product administration beyond RBCs. (b) Not applicable — non-matched cohort.
7	**Variables**	Clearly define all outcomes, exposures, predictors, potential confounders, and effect modifiers. Give diagnostic criteria, if applicable.	Section 2.3 (Intraoperative Fluid Balance) and Section 2.5 (Definition of Flap-Related Complications) — p. 3–4. Exposure: intraoperative fluid balance and crystalloid volume normalized to body weight and operative time (mL/kg/h). Outcomes: flap-related complications (consensus definition cited [2]), total flap loss, suture insufficiency, pneumonia, ICU LOS, in-hospital mortality. Confounders considered: age, sex, comorbidities (PAD, COPD, DM, hypertension, nicotine), operative time, crystalloid volume, urine output, defect characteristics, indication.
8	**Data sources / measurement**	For each variable of interest, give sources of data and details of methods of assessment (measurement). Describe comparability of assessment methods if there is more than one group.	Section 2.2 (Data Collection) — p. 3. Source: electronic medical records (ORBIS, Dedalus HealthCare GmbH, Bonn, Germany) supplemented by paper-based ICU documentation and standardized discharge summaries. Fluid balance calculated according to fixed formula (Section 2.3). Same measurement methods applied to all patients; no group-specific differences.
9	**Bias**	Describe any efforts to address potential sources of bias.	Section 2.7 (Statistical Analysis) — p. 4. Multivariable logistic regression to adjust for age, sex, operative time, crystalloid volume, urine output, defect characteristics and indication. Threshold-based stratification to capture non-linear dose-response. Section 4.2 (Limitations) — p. 7–8 explicitly addresses retrospective design, single-centre setting, chart-review-based outcome ascertainment, residual confounding and evolving practice patterns over the 11-year inclusion period.
10	**Study size**	Explain how the study size was arrived at.	Section 2.1 and Section 3 (Results, opening paragraph) — p. 3 and p. 4.

Item No.	Topic	Recommendation (STROBE)	Reported on (manuscript location)
			All consecutive adult patients meeting inclusion criteria between 2009 and 2020 were included; no a priori sample-size calculation as this was an exploratory analysis of all available cases at a single tertiary centre. Final analytic cohort n = 495; multivariable analysis n = 459 (complete-case).
11	**Quantitative variables**	Explain how quantitative variables were handled in the analyses. If applicable, describe which groupings were chosen and why.	Section 2.3 (Intraoperative Fluid Balance) — p. 3–4. Fluid volumes normalized to body weight and operative time (mL/kg/h) and stratified into pre-specified clinically meaningful categories (≤ 5, > 5–10, > 10–15, > 15–20, > 20 mL/kg/h). Crystalloid volume similarly stratified (≤ 1000, > 1000, > 2000, > 3000, > 4000 mL). Operative time analysed both continuously (per minute, per hour) and categorically (< 4 h, 4–8 h, > 8 h) for sensitivity analysis.
12	**Statistical methods**	(a) Describe all statistical methods, including those used to control for confounding. (b) Describe any methods used to examine subgroups and interactions. (c) Explain how missing data were addressed. (d) Cohort study — If applicable, explain how loss to follow-up was addressed. (e) Describe any sensitivity analyses.	(a) Section 2.7 — p. 4. SPSS v27.0. Shapiro–Wilk for normality. Median [IQR] for non-normal continuous variables. Chi-square / Fisher's exact for categorical; Kruskal–Wallis / Mann–Whitney for continuous. Two-sided p < 0.05. Multivariable logistic regression for flap-related complications and total flap loss; ORs with 95% CI. (b) Subgroup analyses by indication (tumour vs. non-tumour), flap type and defect localization (Sections 3.2, 3.3). (c) Complete-case analysis for multivariable model (n = 459 of 495). (d) Follow-up limited to hospital stay; no loss to follow-up for primary outcomes within this window. (e) Sensitivity analyses with operative time as a categorical variable (< 4 h, 4–8 h, > 8 h; p-trend < 0.001) — Section 3.10, p. 6.
**Results**			
13	**Participants**	(a) Report numbers of individuals at each stage of study—eg, numbers potentially eligible, examined for eligibility, confirmed eligible, included in the study, completing follow-up, and analysed. (b) Give reasons for non-participation at each stage. (c) Consider use of a flow diagram.	(a) Section 3 opening — p. 4. n = 495 included; n = 459 analysed in multivariable models (complete cases). Subgroup of 427 tumour patients (Section 3.3, p. 5). (b) Section 2.1 exclusion criteria — p. 3. (c) Flow diagram not included; can be added as a supplementary figure if requested by reviewers.
14	**Descriptive data**	(a) Give characteristics of study participants (eg, demographic, clinical, social) and information on exposures and potential confounders. (b) Indicate number of participants with missing data for each variable of interest. (c) Cohort study — Summarise follow-up time (eg, average and total amount).	(a) Section 3.1 (Patient Characteristics) and Table 1 — p. 4–5. Age, sex, hypertension, PAD, COPD, diabetes, nicotine use stratified by complication status. (b) Section 3.10 — p. 6. n = 459 of 495 with complete data for multivariable model; missingness predominantly in detailed haemodynamic parameters

Item No.	Topic	Recommendation (STROBE)	Reported on (manuscript location)
			(PPV/SVV) — excluded a priori (Section 2.3, p. 4). (c) Median operative time 480 min (IQR 392–578); median ICU LOS 2 days (IQR 1–5) overall — Section 3.5 and 3.11.
15	**Outcome data**	Cohort study — Report numbers of outcome events or summary measures over time.	Sections 3.1, 3.4, 3.6, 3.7, 3.11 and Tables 2 and 3 — p. 4–7. Overall flap-related complication rate 38.0% (n = 188); total flap loss 17.0% (n = 84); suture insufficiency 5.7% (n = 28); pneumonia rate increased from 8.8% to 31.9% across fluid strata; in-hospital mortality 1.4% overall, up to 28.6% in > 20 mL/kg/h group.
16	**Main results**	(a) Give unadjusted estimates and, if applicable, confounder-adjusted estimates and their precision (eg, 95% CI). Make clear which confounders were adjusted for and why they were included. (b) Report category boundaries when continuous variables were categorized. (c) If relevant, consider translating estimates of relative risk into absolute risk for a meaningful time period.	(a) Section 3.10 (Multivariable Analysis) — p. 6. Adjusted ORs: operative time OR 1.004 per minute (95% CI 1.002–1.006; p < 0.001); crystalloid volume OR 1.19 per litre (95% CI 0.98–1.45; p = 0.076). Adjustment variables (age, sex, operative time, crystalloid volume, urine output) explicitly stated. (b) Category boundaries for fluid (≤ 5, > 5–10, > 10–15, > 15–20, > 20 mL/kg/h) and crystalloid volume (≤ 1000, > 1000, > 2000, > 3000, > 4000 mL) listed in Section 2.3 and Tables 2–3. (c) Adjusted probabilities of flap loss reported: 3% at 4 h, 9% at 8 h, > 20% beyond 12 h — Section 3.10, p. 6.
17	**Other analyses**	Report other analyses done—eg analyses of subgroups and interactions, and sensitivity analyses.	Subgroup analyses: tumour vs. non-tumour patients (Section 3.2); irradiated vs. non-irradiated tumour subgroup (Section 3.3, p. 5); vasopressor users vs. non-users (Section 3.9, p. 6). Sensitivity analysis: categorical operative time analysis confirming dose-response (Section 3.10, p-trend < 0.001).
**Discussion**			
18	**Key results**	Summarise key results with reference to study objectives.	Section 4 (Discussion) opening paragraph — p. 7. Summary: dose-dependent association between intraoperative fluid administration and adverse outcomes; > 10 mL/kg/h threshold for complications, suture insufficiency and pneumonia; U-shaped relationship for flap loss; operative time as independent predictor; prolonged ICU stay and mortality with fluid overload.
19	**Limitations**	Discuss limitations of the study, taking into account sources of potential bias or imprecision. Discuss both direction and magnitude of any potential bias.	Section 4.2 (Limitations) — p. 7–8. Explicitly addressed: (i) retrospective single-centre design; (ii) lack of standardized PPV/SVV documentation; (iii) chart-review-based outcome ascertainment with potential under-reporting; (iv) practice evolution over 11-year inclusion

Item No.	Topic	Recommendation (STROBE)	Reported on (manuscript location)
			period; (v) residual confounding by surgical technique, surgeon experience and patient complexity; (vi) small numbers in highest fluid-rate categories limiting precision at extremes; (vii) threshold-based analysis does not capture continuous dose-response.
**20**	**Interpretation**	Give a cautious overall interpretation of results considering objectives, limitations, multiplicity of analyses, results from similar studies, and other relevant evidence.	Section 4 (Discussion paragraphs 2–11) — p. 7. Findings contextualized against prior cohort studies (Burkhard [3], Dooley [4], Pattani [5], Booi [30], Rhee [6]), GDFT trials (Tapia [16], Lahtinen [9]), GDFT meta-analyses (Chong [12], Sun [33]), mechanistic evidence (Sukudom [17] — glycocalyx), and the Cologne anticoagulation cohort (Torabi [7]). Editorial controversies around fluid composition explicitly discussed [13,14,18,25,26].
**21**	**Generalisability**	Discuss the generalisability (external validity) of the study results.	Section 4.2 (Limitations) and Section 4.3 (Future Directions) — p. 7–8. Single-centre European tertiary-care setting; head and neck free-flap reconstructions predominate; results should be confirmed in multi-centre prospective settings before broad generalization to other reconstructive populations or healthcare systems.
**Other Information**			
**22**	**Funding**	Give the source of funding and the role of the funders for the present study and, if applicable, for the original study on which the present article is based.	"Funding" section — p. 8. Open-access funding enabled and organized by Projekt DEAL; Article Processing Charge supported by Deutsche Forschungsgemeinschaft (DFG, German Research Foundation, grant 491454339). No role of the funder in study design, data collection, analysis, interpretation or manuscript preparation. No competing interests (Conflicts of Interest declaration, p. 8).

Note: Page-number references correspond to the manuscript file *Torabi_FluidTherapy_FreeFlap_JCM_FinalRevised_v2.docx*. Numbers in square brackets refer to the manuscript reference list.

The STROBE checklist is available at *https://www.strobe-statement.org*. An explanation and elaboration article discusses each checklist item and gives methodological background and published examples of transparent reporting: Vandenbroucke JP et al. *PLoS Med*. 2007;4(10):e297. Information on the STROBE Initiative is available at www.strobe-statement.org.
